# Effects of home-based respiratory muscle training in children and adolescents with chronic lung disease* **

**DOI:** 10.1590/S1806-37132014000600006

**Published:** 2014

**Authors:** Iván Rodríguez, Daniel Zenteno, Carlos Manterola

**Affiliations:** San Sebastian University, Faculty of Health Science, Center of Molecular Medicine, Concepción, Chile. Center of Molecular Medicine, Faculty of Health Science, San Sebastian University. Concepción. Chile; Guillermo Grant Benavente Hospital, Department of Pediatrics, Concepción, Chile. Department of Pediatrics, Guillermo Grant Benavente Hospital, Concepción, Chile; Autonomous University, Center for Biomedical Research, Temuco, Chile. Center for Biomedical Research, Autonomous University, Temuco, Chile

**Keywords:** Breathing exercises, Cystic fibrosis, Bronchiolitis obliterans, Neuromuscular diseases, Cough

## Abstract

**OBJECTIVE:**

Respiratory muscle weakness is a functional repercussion of chronic lung disease (CLD). The objective of this study was to assess the effects of home-based respiratory muscle training (RMT) in children and adolescents with CLD or neuromuscular disease (NMD).

**METHODS:**

This was a quasi-experimental study involving children and adolescents with CLD or NMD. Before and after 6 months of home-based RMT, we measured respiratory muscle strength (MIP and MEP), PEF, and peak cough flow (PCF). We made statistical comparisons between the pre-RMT and post-RMT values, as well as evaluating the correlation between the duration and effect of RMT.

**RESULTS:**

The study included 29 patients, with a mean age of 12 years (range, 5-17 years), of whom 18 (62.1%) were male. The CLD group comprised 11 patients (37.9%), and the NMD group comprised 18 (62.1%). The mean duration of the RMT was 60 weeks (range, 46-90 weeks) in the CLD group and 39 weeks (range, 24-89 weeks) in the NMD group. In comparison with the pre-RMT values, the post-RMT values for MIP and MEP were significantly higher in both groups, whereas those for PEF and PCF were significantly higher only in the NMD group. We found no correlation between the duration and the effect of RMT.

**CONCLUSIONS:**

Home-based RMT appears to be an effective strategy for increasing respiratory muscle strength in children and adolescents with CLD or NMD, although it increased the ability to cough effectively only in those with NMD.

## Abstract (pt)

**OBJETIVO:**

A fraqueza muscular respiratória é uma repercussão funcional da doença pulmonar crônica (DPC). O objetivo deste estudo foi avaliar os efeitos do treinamento muscular respiratório (TMR) domiciliar em crianças e adolescentes com DPC ou doença neuromuscular (DNM).

**MÉTODOS:**

Estudo quasi-experimental com crianças e adolescentes com DPC ou DNM. Foram medidos a força muscular respiratória (PEmáx e PImáx) e o pico de fluxo da tosse (PFT) antes e depois de 6 meses de TMR domiciliar. Foram realizadas comparações estatísticas entre valores pré- e pós-TMR e foram avaliadas as correlações entre a duração e o efeito do TMR.

**RESULTADOS:**

Foram incluídos no estudo 29 pacientes, com média de idade de 12 anos (variação, 5-17 anos), dos quais 18 (62,1%) eram meninos. O grupo DPC consistiu em 11 pacientes (37,9%) e o grupo DNM, em 18 (62,1%). A média da duração do TMR foi de 60 semanas (variação, 46-90 semanas) no grupo DPC e de 39 semanas (variação, 24-89 semanas) no grupo DNM. Em comparação com os valores pré-TMR, os valores pós-TMR para PImáx e PEmáx foram significativamente maiores nos dois grupos, enquanto aqueles para PFE e PFT foram significativamente maiores apenas no grupo DNM. Não houve correlações entre a duração e o efeito do TMR.

**CONCLUSÕES:**

O TMR domiciliar parece ser uma estratégia eficaz para o aumento da força muscular respiratória em crianças e adolescentes com DPC ou DNM, embora aumente efetivamente a capacidade de tosse somente naqueles com DNM.

## Introduction

Respiratory muscle weakness is one of the main functional repercussions caused by the deterioration of lung function in children with chronic lung disease (CLD) or neuromuscular disease (NMD).^(^ 1 ^-^ 3 ^)^ Such weakness leads to the development of alveolar hypoventilation, the formation of microatelectasis, and dysfunction of the cough mechanism, factors that increase the risk of respiratory failure.^(^ 4 ^)^

It has been shown that respiratory muscle training (RMT) is an effective strategy to mitigate losses in respiratory muscle strength and endurance. ^(^ 5 ^-^ 7 ^)^ However, there is no evidence-based consensus regarding the protocols and ideal training methods to obtain significant improvements according to the physiopathological characteristics of specific diseases, especially in children and adolescents. In Chile, it is recommended that RMT, of progressive duration and with loads of 20-30% of the MIP, be performed periodically in children and adolescents with CLD.^(^ 8 ^)^

Patient adherence to rehabilitation is crucial to achieving the objectives, and home-based rehabilitation protocols have produced positive results.^(^ 9 ^)^ This is because the rehabilitation is undertaken in a familiar setting, without the need to go to a health care clinic, thus facilitating the attainment of the rehabilitation goals. 

There have been few reports on the effects of unsupervised RMT on lung function. Therefore, the aim of this study, conducted in Chile, was to evaluate the effects that a home-based RMT program has on lung function in children and adolescents with CLD or NMD.

## Methods

### Design

This was a quasi-experimental study that included the respiratory function records collected before and after the implementation of a 6-month home-based RMT protocol for patients with CLD or NMD participating in the pediatric respiratory rehabilitation program at Guillermo Grant Benavente Hospital, in the city of Concepción, Chile.

### Participants

Through non-probabilistic convenience sampling of prevalent and incident cases, we selected NMD and CLD patients between 6 and 18 years of age who had begun the home-based RMT protocol between May of 2011 and May of 2013. We included only those patients who had participated in the protocol for at least 6 months, although those who had continued the protocol for more than 6 months were also included. Patients with cognitive deficits were excluded, as were those whose parents or legal guardians did not allow them to participate in the study.

### Sample size calculation

The sample size was calculated considering a 5% risk of a type I error, a power of 85%, a minimum expected difference in MIP of 27 cmH_2_O, a variance of 436.81, and two study groups (CLD and NMD).^(^ 5 ^)^ We thus estimated the minimum sample size required to be 10 subjects per group.

### Procedures

For each of the patients, we collected data related to medical history, together with baseline data for weight, height, age, gender, spirometry results, MIP, MEP, PEF, and peak cough flow (PCF), as well as the number of weeks participating in the home-based RMT protocol. We also collected post-RMT values for MIP, MEP, PEF, and PCF. All variables were measured by the same evaluator.

Spirometry was performed with a Microlab ML3500 spirometer (Micro Medical Ltd, Rochester, England) according to the guidelines established by the American Thoracic Society. We recorded values for FEV_1_, FVC, the FEV_1_/FVC ratio, and FEF_25-75%_, in absolute values and in the percentage of the predicted value, according to Knudson et al.^(^ 10 ^)^

As a surrogate for respiratory muscle strength, we evaluated MIP, measured during a maximum inspiratory effort, from residual volume, maintained for at least one second, and MEP, measured during a maximum expiratory effort, from TLC, maintained for at least one second. We used a vacuum/pressure gauge (NS 120-TRS; Instrumentation Industries Bethel Park, PA, USA), calibrated in centimeters of water (from 0 to −120 cmH_2_O and from 0 to +120 cmH_2_O). The MIP and MEP values are expressed in absolute values and percentage of the normal value according to the reference values for age and gender, as determined by Szeinberg et al.^(^ 11 ^)^

The PEF and PCF were measured with a peak flow meter (Mini-Wright^(r)^; Clement Clarke International, Essex, England). The PEF was evaluated with the subject seated, wearing a nose clip, and the indication was given to blow with the greatest possible force from TLC. The test was performed a maximum of eight times, and we recorded the highest reproducible value in three attempts with a difference no greater than 10% between each value. The PCF was evaluated with the subject seated, and the indication was given to inhale to TLC, then to perform a maximum coughing maneuver through the same instrument. The results are expressed in liters per minute.

### RMT protocol

The RMT protocol involved the use of threshold load valves (IMT or PEP; Philips Respironics, Murrysville, PA, USA), once a day for at least 5 days a week and for at least 6 months. Every patient, as well as their parents or caregivers, were trained in the methodological aspects of the RMT protocol and in strategies to maintain adequate adherence in the home setting. The patients were evaluated once every 4-6 weeks, at which times they were reminded of the importance of adhering to the RMT protocol. The daily inspiratory muscle training consisted of 3 series of 3 min of ventilation on demand through the valve, at a load of 30-50% of the MIP, with a 1-min rest period between each series. The daily expiratory muscle training consisted of 3 series of 15 exhalations through the valve, at a load of 30-50% of the MEP, with a 1-min rest period between each series.

### Ethical considerations

The parents or legal guardians of all participating patients gave written informed consent. Patients over the age of 12 also signed the informed consent form. The research protocol was approved by the Research Ethics Committee of Guillermo Grant Benavente Hospital.

### Statistical analysis

Using the Statistical Package for the Social Sciences, version 11.5 (SPSS Inc., Chicago, IL, USA), we performed an exploratory analysis of the data, after which we calculated descriptive statistics, including means, ranges, and proportions. Because the variables showed non-normal distribution, we applied analytical statistics, using the Wilcoxon signed rank test for paired comparisons in order to evaluate the difference between the pre-RMT and post-RMT values, whereas we used the Mann-Whitney U test to compare means between the two groups. In addition, we used Spearman's rho correlation coefficient to determine whether the duration of RMT correlated with increases in the variables MIP, MEP, PEF, and PCF. Values of p < 0.05 were considered statistically significant.

## Results

The study included 29 patients, with a mean age of 12 years (range, 5-17 years), of whom 18 (62.1%) were male. The CLD group comprised 11 patients (37.9%), diagnoses including post-infectious bronchiolitis obliterans (n = 3), cystic fibrosis (n = 5), and bronchiectasis (n = 3). The NMD group comprised 18 (62.1%), diagnoses including Duchenne muscular dystrophy (DMD, n = 7), spinal muscular atrophy (SMA, n = 3), myelomeningocele (n = 2), facioscapulohumeral muscular dystrophy (n = 1), Becker muscular dystrophy (n = 1), Bethlem myopathy (n = 1), congenital myopathy (n = 1), Charcot-Marie-Tooth disease (n = 1), and Guillain-Barré syndrome (n = 1). 

The pulmonary function tests revealed that all of the patients in the CLD group presented an obstructive pattern, the impairment being minimal in two cases, slight in three, moderate in one, and severe in five. In the NMD group, we observed a restrictive pattern in ten cases (slight impairment in six; moderate impairment in two; and severe impairment in two) and an obstructive pattern (with minimal impairment) in one case. The remaining patients showed no spirometric alterations. Table 1 shows the characteristics of the sample at baseline, and Table 2 shows the patient distribution by diagnosis.

**Table 1 -:** Baseline characteristics of the patient sample.a

Characteristic	NMD group	CLD group
	(n = 18)	(n = 11)
Age (years)	12 (5-17)	13 (5-16)
Male/female, n/n	11/6	6/5
Weight (kg)	41 (23-88)	38 (18-71)
Height (m)	1.45 (1.10-1.60)	1.42 (1.03-1.61)
Ambulatory/non-ambulatory, n/n	8/10	11/0
Duration of RMT (weeks)	39 (24-89)	60 (46-90)
Lung function		
FEV_1_ (L)	1.67 (0.49-2.95)	1.35 (0.55-2.67)
FEV_1_ (% of predicted)	79.0 (30-116)	54.0 (30-99)
FVC (L)	1.85 (0.58-3.28)	1.92 (0.65-3.43)
FVC (% of predicted)	78.5 (11-114)	81.0 (49-112)
FEV_1_/FVC ratio	88.5 (71-100)	64.0 (39-85)
FEF_25-75%_ (L/sec)	1.83 (0.5-5.9)	0.57 (0.26-2.29)
FEF_25-75%_ (% of predicted)	66.0 (30-142)	31.0 (11-72)

NMD: neuromuscular disease; CLD: chronic lung disease; and RMT: respiratory muscle training.

a Results are expressed as mean (range), except where otherwise indicated.

**Table 2 -:** Patient distribution by diagnosis (N = 29).

Diagnosis	Distribution
Neuromuscular diseases	
Duchenne muscular dystrophy	7
Facioscapulohumeral muscular dystrophy	1
Becker muscular dystrophy	1
Type II spinal muscular atrophy	2
Type III spinal muscular atrophy	1
Bethlem myopathy	1
Myelomeningocele	2
Congenital myopathy	1
Charcot-Marie-Tooth disease	1
Guillain-Barré syndrome	1
Chronic lung diseases	
Post-infectious bronchiolitis obliterans	3
Cystic fibrosis	5
Bronchiectasis	3

In order to maintain adherence to the training protocol, patients were evaluated once every 4-6 weeks by a physiotherapist specializing in respiratory rehabilitation, who refreshed the memory of the patients regarding the methodological aspects of the RMT. All the patients continued the RMT for a period longer than the prescribed 6 months, the mean duration of RMT being 39 weeks (range, 24-89 weeks) in the NMD group and 60 weeks (range, 46-90 weeks) in the CLD group. 

**Figure 1 -:**
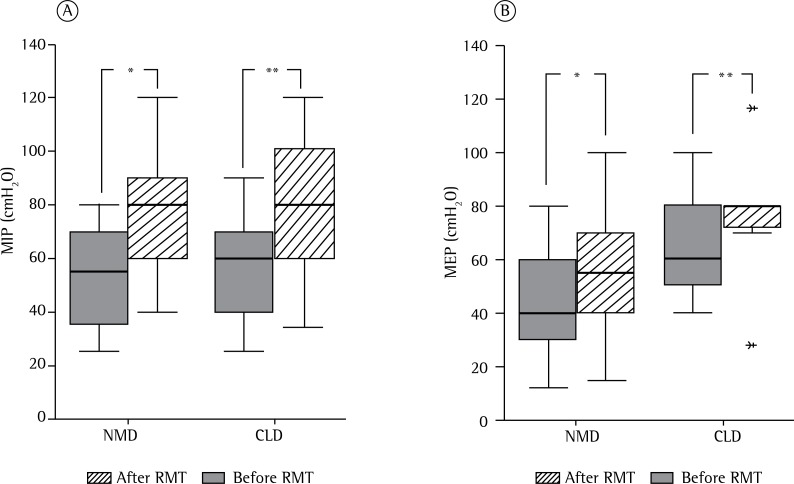
Effect of respiratory muscle training (RMT) on MIP (A) and MEP (B) in children and adolescents with chronic lung disease (CLD; n = 11) or neuromuscular disease (NMD; n = 18). *p < 0.05 (Wilcoxon test for paired comparisons). **p < 0.01 (Wilcoxon test for paired comparisons).

**Figure 2 -:**
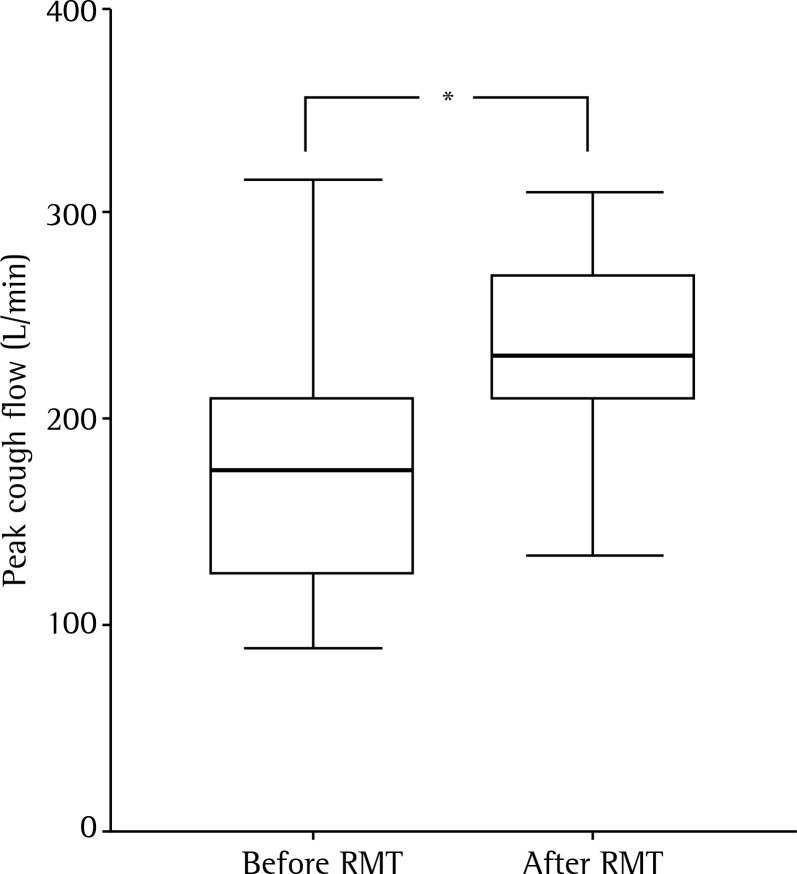
Effect of respiratory muscle training (RMT) on peak cough flow in children and adolescents with neuromuscular disease. *p < 0.05 (Wilcoxon signed rank test for paired comparisons).

In the NMD group, there was a significant post-RMT increase in respiratory muscle strength, as evidenced by the mean improvements (over baseline) in MIP (an increase in the absolute value of 25 cmH_2_O [45.4%; p = 0.004], as well as an increase in the percentage of the predicted value [from 44.5% {range, 22.9-90.0%} to 63.0% {range, 30.7-100%}; p = 0.01; Figure 1A]) and in MEP (an increase in the absolute value of 15 cmH_2_O [37.5%; p = 0.007], as well as an increase in the percentage of the predicted value [from 28% {range, 9-48%} to 33% {range, 11-62%}; p = 0.002; Figure 1B]). In the CLD group, there were similar post-RMT improvements in the mean MIP values (an increase in the absolute value of 20 cmH_2_O [33.3%; p = 0.01], as well as an increase in the percentage of the predicted value [from 47.5% {range, 19-82%} to 73% {range, 32-110%}; p = 0.005; Figure 1A]) and in the mean MEP values (an increase in the absolute value of 20 cmH_2_O [33.3%; p = 0.02], as well as an increase in the percentage of the predicted value [from 39% {range, 28-74%} to 48% {range, 27-85%}; p = 0.021; Figure 1B]). Table 3 shows the variations between the pre- and post-RMT evaluations, by group. As can be seen there (and in Figure 2), the mean PEF increased by 85 L/min (56.6%) in the NMD group (p = 0.001), compared with only 25 L/min (14.2%) in the CLD group (p = ns), whereas the mean PCF increased by 55 L/min in the NMD group (31.4%; p = 0.001), compared with only 16 L/min (8.5%) in the CLD group (p = ns). We found no correlation between the duration and the effect of the RMT, in either group.

**Table 3 -:** Effects of respiratory muscle training on respiratory muscle strength, peak expiratory flow, and peak cough flow.

Variable	NMD group (n = 18)	CLD group (n = 11)
∆MIP (cmH_2_O)	+25 (45.4%)*	+20 (33.3%)**
∆MEP (cmH_2_O)	+15 (37.5%)*	+20 (33.3)**
∆PEF (L/min)	+85 (56.6%)*	+25 (14.2%)
∆PCF (L/min)	+55 (31.4%)*	+16 (8.5%)

?: Variation between baseline values and those obtained after 6 months of respiratory muscle training; NMD: neuromuscular disease; CLD: chronic lung disease; and PCF: peak cough flow.

* p < 0.01 (Wilcoxon signed rank test for paired comparisons).

** p < 0.05 (Wilcoxon signed rank test for paired comparisons).

## Discussion

In the present study, we found that, in children and adolescents with NMD or CLD, unsupervised home-based RMT for at least 6 months produced a significant increase in respiratory muscle strength (i.e., in MIP and MEP). In addition, we observed significant post-RMT improvements in PEF and PCF in the patients with NMD. However, no correlation was identified between the duration of the RMT and the magnitude of the improvements in pulmonary function.

Our results are consistent with those reported by Koessler et al.,^(^ 5 ^)^ who evaluated the effects of a two-year home-based RMT protocol in patients with DMD or SMA.^(^ 12 ^-^ 16 ^)^ The authors observed that RMT increased inspiratory muscle strength and endurance by 49% and 16%, respectively. Those values were obtained after 10 months of training, the point at which a plateau was reached in terms of the increase in strength.^(^ 5 ^)^ In the DMD patients evaluated in the present study, we observed post-RMT increases in the mean absolute values for MIP and MEP, which increased by 30 cmH_2_O (60%) and 10 cmH_2_O (33%), respectively, compared with 25 cmH_2_O (47%) and 6.7 cmH_2_O (14%), respectively, in our SMA patients. In the remaining NMD group patients, the post-RMT increases in MIP and MEP ranged from 8 cmH_2_O to 65 cmH_2_O and from 10 cmH_2_O to 48 cmH_2_O, respectively, the exceptions being the patient with Becker muscular dystrophy, who showed no changes in MIP or MEP, and the patient with congenital myopathy, who showed no change in MIP.

It is of note that 66% of our patients with NMD (n = 12) were reassessed after 35 weeks of training, and the overall magnitude of the increase in the MIP recorded was 45.4%. It is likely that, at this stage, inspiratory muscle strength would have reached the plateau phase, and that subsequent evaluations would show only modest increases in the MIP. 

The PEF and PCF are parameters that can objectively quantify cough function, as well as being directly associated with respiratory muscle strength.^(^ 17 ^,^ 20 ^)^ In this context, it has been shown that PCF levels below 160 L/min translate to ineffective cough and constitute a risk factor for the development of respiratory diseases.^(^ 19 ^,^ 20 ^)^ There have been few studies evaluating the effect of RMT on cough function in patients with NMD. Expiratory muscle training protocols have been shown to improve cough function in patients with multiple sclerosis.^(^ 21 ^,^ 22 ^)^ In a recent study, Aslan et al.^(^ 23 ^)^ evaluated the effect of RMT on pulmonary function variables (including PCF) in patients with slowly progressive NMD. The authors observed significant post-RMT increases only in respiratory muscle strength. In all of the patients with NMD evaluated in our study, PEF and PCF both increased significantly after RMT. Among our patients with DMD, we observed significant post-RMT increases in the mean absolute values for PEF and PCF, which increased by 90 L/min (60%) and 50 L/min (26%), respectively, compared with 35 L/min (22%) and 96 L/min (70%), respectively, among our patients with SMA. In the remaining NMD group patients, the post-RMT increases in PEF and PCF ranged from 30 L/min to 235 L/min and from 20 L/min to 150 L/min, respectively. It is noteworthy that, of the 18 NMD group patients, 8 (44.4%) presented a pre-RMT value of PCF below 160 L/min and 6 of those patients managed to increase their PCF to above 160 L/min after the RMT.

Data in the literature are inconsistent regarding the effects of RMT in patients with CLD. In patients with adenovirus sequelae, RMT has been shown to increase respiratory muscle strength.^(^ 24 ^)^ Houston et al.^(^ 7 ^)^ showed that RMT increased the strength and endurance of the respiratory muscles in patients with cystic fibrosis. However, the authors found no such improvement in lung flows and volumes, and they suggested that their results be confirmed in studies with a higher level of methodological quality.^(^ 7 ^)^ Santana-Sosa et al.^(^ 25 ^)^ observed that RMT combined with general muscle training had beneficial effects for respiratory muscle strength and physical conditioning in children with cystic fibrosis.^(^ 25 ^)^ In the CLD group, there was an overall post-RMT increase in respiratory muscle strength (i.e., MIP and MEP) of 33%, the greatest improvement being observed in our cystic fibrosis patients, who showed increases of 30 cmH_2_O (42%) and 25 cmH_2_O (45%) in MIP and MEP, respectively. In contrast, our patients with post-infectious bronchiolitis obliterans showed an increase in MIP of only 20 cmH_2_O (33.3%) and our patients with bronchiectasis showed an increase in MEP of only 25 cmH_2_O (50%). None of our CLD patients showed significant post-RMT changes in PEF or PCF. That was to be expected, because all of those patients presented an obstructive pattern in the spirometry tests, with FEF_25-75%_ levels below the predicted value, which presumably negates any possible gains in cough function achieved from the RMT, as has been reported in the literature.^(^ 7 ^,^ 19 ^)^

The present study has some limitations. We divided our patient sample into two groups (NMD and CLD) in order to evaluate the effect of RMT according to the origin of the pulmonary impairment, allowing us to obtain a broader view of the potential benefits of this rehabilitation strategy. However, due to heterogeneity of the sample and the small sample size, it was not possible to perform statistical analyses of the subgroups (i.e., by diagnosis), which prevented us from drawing conclusions and making recommendations for the management of specific conditions. In addition, although the duration of RMT was greater among our patients with CLD than among those with NMD, it was not possible to show interdependence between the duration and the effects of RMT, only a weak (non-significant) correlation being observed between the two, in both groups (rho < 0.2; p ≥ 0.05). The lack of direct follow-up of the RMT protocol is another limitation of our study, because it was not possible to verify the level of patient adherence to the protocol. Nevertheless, in the periodic evaluations, we were able to establish that all of the patients knew how to implement the protocol as they had been taught in the first session. Furthermore, in interviews with the parents and caregivers, we determined that all of the patients engaged in the training at least 5 days a week, although that data might have been subject to a reporting bias (embarrassment leading parents and caregivers to overestimate the level of adherence). Moreover, considering that all the assessment tests require patient cooperation, there was no control group. Therefore, it was not possible to rule out the possibility of a measurement bias, which could have resulted in the overestimation of the magnitude of the post-RMT increases in the variables evaluated. These potential sources of bias could reduce the validity of the outcomes. 

We can conclude that home-based RMT, if continued for at least 6 months, is an effective strategy to improve respiratory muscle strength in patients with NMD or CLD, mainly in those with cystic fibrosis. Specifically in patients with NMD, this rehabilitation strategy also appears to effect significant increases in the determinants of cough function, such as PEF and PCF.
